# A unified model of the standard genetic code

**DOI:** 10.1098/rsos.160908

**Published:** 2017-03-01

**Authors:** Marco V. José, Gabriel S. Zamudio, Eberto R. Morgado

**Affiliations:** 1 Theoretical Biology Group, Instituto de Investigaciones Biomédicas, Universidad Nacional Autónoma de México, MexicoD.F. 04510, Mexico; 2 Facultad de Matemática, Física y Computación, Universidad Central ‘Marta Abreu’ de Las Villas, Santa Clara, Cuba

**Keywords:** standard genetic code, symmetry groups, aminoacyl-tRNA synthetases, group actions, automorphisms, polar requirement

## Abstract

The Rodin–Ohno (RO) and the Delarue models divide the table of the genetic code into two classes of aminoacyl-tRNA synthetases (aaRSs I and II) with recognition from the minor or major groove sides of the tRNA acceptor stem, respectively. These models are asymmetric but they are biologically meaningful. On the other hand, the standard genetic code (SGC) can be derived from the primeval RNY code (R stands for purines, Y for pyrimidines and N any of them). In this work, the RO-model is derived by means of group actions, namely, symmetries represented by automorphisms, assuming that the SGC originated from a primeval RNY code. It turns out that the RO-model is symmetric in a six-dimensional (6D) hypercube. Conversely, using the same automorphisms, we show that the RO-model can lead to the SGC. In addition, the asymmetric Delarue model becomes symmetric by means of quotient group operations. We formulate isometric functions that convert the class aaRS I into the class aaRS II and vice versa. We show that the four polar requirement categories display a symmetrical arrangement in our 6D hypercube. Altogether these results cannot be attained, neither in two nor in three dimensions. We discuss the present unified 6D algebraic model, which is compatible with both the SGC (based upon the primeval RNY code) and the RO-model.

## 1. Introduction

The insight that all organisms on Earth are related by common descent [1] is a remarkable scientific achievement. Indeed, the Last Universal Common Ancestor seemed to obey already the standard genetic code (SGC), which is *nearly universal*. The problem of the origin and evolution of the SGC is a fundamental challenge in biology. After the decipherment of the SGC [2], there have been several proposals that account for both the origin and evolution of the genetic code [3–11]. There seems to be a consensus that the SGC conserves vestiges of earlier codes, to wit, the operational [12,13] and anticodon codes [14,15]. The amino acid specific aminoacylation of tRNAs (operational code) is localized in the acceptor stem of the tRNAs and is recognized by the corresponding aminoacyl-tRNA synthetases (aaRSs) [12,13]. Indeed, most living organisms still contain relics of these primeval codes, which are a palimpsest over which the evolving codes were later additions in order to arrive at the frozen SGC [16,17]. In fact, the primeval RNY code was already frozen [18].

The SGC is written in an alphabet of four letters (C, A, U, G), grouped into words three letters long, called triplets or codons. In general, and in most textbooks, the genetic code is represented in a two-dimensional (2D) table arranged in such a way that it is possible to readily find any amino acid from the three letters, written in the 5′ to 3′ direction of the codon [4,19,20]. Each of the 64 codons specifies one of the 20 amino acids or else serves as a punctuation mark signalling the end of a message. The standard table of codon assignments derives from the obvious representation of the triplet code as a 4 × 4 × 4 cube. Three-dimensional (3D) algebraic models using a Galois Field of four elements GF(4) [21,22] or Lie algebras [22] have also been formulated. More revealing representations have been attained using the six-dimensional (6D) hypercube [23,24] of the 64 codons of the SGC. Observing that 64 is equal not only to 4^3^ but also to 2^6^, the codon table can be organized as a 6D hypercube or 6D vector space ${\left(Z_{2}\right)}^{6}$ over the binary field $Z_{2}=\{0,1\}$ [24]. The phenotypic graphs of amino acids have been obtained from the topology of the SGC [15]. Additionally, circular representations of the SGC have been proposed [25–27]. Given 64 codons and 20 amino acids plus a punctuation mark, there are $21^{64}\approx 4\times 10^{84}$ possible genetic codes. The result that only one in every million random alternative codes is more efficient than the SGC [28] implies that there could be approximately 4 × 10^78^ genetic codes as efficient as the SGC. This calculation does not offer deeper insights concerning the origin and structure of the SGC, particularly the frozen accident. Francis Crick [4] argued that the SGC need not be special at all; it could be nothing more than a ‘frozen accident’. Yet as we show in this article, there are indeed several features that are special about the SGC: firstly, it can be partitioned *exactly* into two classes of aaRRs in six dimensions; secondly, it displays symmetry groups when the polar requirement (PR) is used; and thirdly, the SGC can be broken down into a product of simpler groups reflecting the pattern of degeneracy observed, and the salient fact that evolution did not erase its own evolutionary footsteps.

The search for symmetries in the SGC has been made by examining the tRNA [29,30] and aaRSs [3,6–8], using phylogenetic methods [31,32]. Less popular have been algebraic models seeking to unveil hidden symmetries of the SGC [33,34]. For example, the SGC has been theoretically derived from a primeval RNY (R means purines, Y pyrimidines, and N any of them) genetic code [9] under a model of sequential symmetry breakings [16,21,35]. Universal vestiges of these evolutionary steps were found in current genomes of both Eubacteria and Archaea [35]. The SGC is implemented via the tRNAs that bind each codon with its anticodon. These molecules define the genetic code, by linking the specific amino acids and tRNAs with the corresponding anticodons [7]. The tRNA molecule itself displays two codes, the operational code and the anticodon code. Typically, two genetic codes are considered, to wit, the ‘classic’ code represented in tRNA by an anticodon for reading codons in mRNA, and the other is the ‘second’ [12] operational RNA code [13,36] mapped mainly to the acceptor for appropriate aminoacylation at its 3′ terminus. In addition, there are also two separate codes, embedded in the tRNA anticodon and acceptor-stem bases that correspond, respectively, to amino acid size and hydrophobicity [37,38]. These coding elements evolved separately and independently [38]. The earlier appearance of an acceptor-stem code, before the emergence of the universal genetic code [13] is supported experimentally by (i) the reciprocal biochemistry of minihelix acylation by full-length synthetases [39] and (ii) the acylation of full-length tRNAs by truncated synthetases called Urzymes [40].

PR is an abiotic feature of free amino acids in solution. PR is a physico-chemical property of each amino acid, defined by their migration in paper chromatographic experiments in aqueous solutions of nucleobases [41]. PR is directly related to the organization of the codon table and its amino acids [42]. In addition, PR is related to the partition of amino acid in a polar–non-polar interface [43]. The SGC is also robust to errors of single base mutations and this is reflected when PR is used as a metric of amino acid similarity [28,44,45]. Moreover, the phenotypic graphs of amino acids exhibit disjoint clusters of amino acids when their PR values are used [15]. The genetic code became optimized with respect to PR. By observing the microscopic environments of the amino acids in binary solution, it is apparent that the PR is related to how an amino acid partitions across a polar–non-polar interface. Several theoretical studies have found a high degree of error tolerance in the genetic code when PR is used as a measure of amino acid similarity [28,45–47]. Polar–non-polar interfaces may have played a role in the establishment or development of the early genetic code. It is highly improbable that the genetic code became optimized with respect to PR purely by chance.

As far as translation is concerned, it does not make sense to consider one code without the other. The present-day operational code is intricately carved in the structure of tRNA acceptors and cognate aaRSs, whereas the anticodon code is reduced to codon–anticodon interactions. The catalytic proteins required to accelerate this binding are divided between two very ancient enzyme superfamilies, the class I and class II aaRSs, each activating 10 of the 20 canonical amino acids [8]. The present correspondence of the two codes is provided by 20 specific aaRSs divided into two strikingly dissimilar classes of 10 members each. There are only 20 aaRSs, one for each amino acid (and, respectively, for isoacceptor tRNAs); hence, the operational code is non-degenerate [12]. Such a non-degeneracy, inherent only to the acceptor code, may indicate the historically subsidiary role of anticodons in aminoacylation. Otherwise, more than 20 aaRSs could exist, one for each anticodon rather than one for each amino acid. The two aaRSs recognize the acceptor helix from opposite sides: class I aaRS approaches the helix from the side of its minor groove and attaches the amino acid to the 2′OH group of the terminal adenosine ribose, while class II aaRS approaches from the side of major groove and attaches the amino acid to the 3′OH group [8]. The aaRSs are divided into two classes distinguished by their structures [8]. The term ‘class’ is used to distinguish both the enzymes and the amino acids that they activate [8]. Polarity and size are used to distinguish between the two classes of amino acids [37,39]. Class II amino acids occur significantly more frequently at the surfaces of proteins, whereas class I amino acids occur more frequently in their cores [39]. Notably, the two synthetases classes seem to have descended from ancestors coded by opposite strands of the same gene [48]. There is no need for the aaRS to recognize the anticodon in order to properly aminoacylate the tRNA. This means that the two codes coevolved right at the origin of translation. This encoding system seems now lost in the dimness of the past. Rodin & Ohno [49] found that the two families of aaRSs exhibit significant sequence similarity, but only when their coding sequences are compared in the opposite direction. This finding prompted Rodin & Ohno [49] to suggest that the two synthetases families originated as two-protein coding genes located on the complementary strands of the same primordial double-stranded RNA. Assuming that the partition into two mechanisms of tRNA-aminoacylation is a relic that dates back to the primordial genetic code in the RNA world, Delarue [3] proposed a simple model based upon successive binary choices for the assignment of codons to amino acids. Both Delarue's [3] and Rodin & Rodin's [7] models reorganize the codon table to reflect these contrasting molecular recognition modes by the two aaRS classes. These authors propose that this dual complementarity is frozen from an earlier stage in the code's development, at which triplet reading frames had been established, but only the middle bases of the anticodons had been fixed, perhaps coinciding with the second step of Delarue's differentiation genealogy [7]. They concluded that new codons were recruited in pairs, because translation of both sense and antisense strands would require that meaning be attached to both codons and their anticodons. We chose these models in order to prove the power of algebraic methods to understand each model and because our approach facilitates the comparison of the predictions among different models. In particular, the RO-model has a sound experimental background [37–40,48].

Herein the RO [7,49,50] and Delarue (D) [3] models for the origin of the genetic code are analysed in terms of its symmetrical properties. The RO- and D-models are asymmetrical. In this work, we assume a primeval RNY code [9], and make the same assumption of the RO-model, i.e. that the SGC can be divided according to the two classes of aaRSs I and II. We formulate isometries with which we arrive precisely to our symmetrical algebraic model [15,21,35].

The article is organized as follows. We start with some basic definitions of group theory and we provide the definition of the group action over the set of nucleotides. Then, we analyse the Rodin–Rodin model [50] of dividing the table of the genetic code according to the two classes of aaRSs. This table is symmetric but it is biologically incorrect. Then, we formulate simple isometric transformations that allow us to transform the RO-model which is asymmetric but biologically correct, into the SGC model based on the primeval RNY code and vice versa. We define an automorphism that converts the class aaRS I into the class aaRS II. We also model the asymmetric D-model into a symmetrical one by means of quotient groups. As a direct application of the 6D model of the SGC, we used the four scales of PR of each amino acid [41] and it neatly divides the SGC into four symmetrical groups. Finally, we discuss the results in terms of our model, which is compatible with the RO- and D-models and the primeval RNY code [9]. In other words, we have a unique 6D model, which is consistent with the RNY primeval genetic code and with the distribution of the two classes of aaRS.

## 2. Mathematical background

Group theory is a branch of abstract algebra that deals with the notion of symmetry of a geometrical object, making the set of symmetries of an object a group structure.

### 2.1. Definition of a group

A group is a set G with a binary operation ○ that combines any two elements of G and returns an element in G. This ordered pair is denoted as (G,∘) which satisfies the following properties:

1. Closure: For all *a,b* in G, the resulting element is also in G_._
2. Associativity: For all *a,b,c* in G, the next equality holds: (a∘b)∘c=a∘(b∘c).
3. Identity element: There exists an element *e* in G such that a∘e=e∘a=a for all *a* in G.
4. Inverse element: For all *a* in G, there exists an element *a*′ such that $a\circ a^{\prime }=a^{\prime }\circ a=e$, where *e* is the identity element.

### 2.2. Definition of a group action

If G is a group and X is a set then a group action is a function f:G×X→X, (a,x)→a∗x that satisfies the following axioms:

1. Compatibility: For all *a,b* in G and all x in X the equality (a ∘b)∗x=a∗(b ∗x) holds.
2. Identity: For all x in X, e ∗x=x, where *e* is the identity element of G.

Then, it is said that G acts on X and X is a G− set.

A group action is the description of symmetries of an object using an external group. The essential elements of the object are described in a set and the operating group is known as the group of symmetries and its members correspond to some of the one-to-one transformations of the set. When considering a point x∈X and the group G operating over X, the set Gx={g∗x|g∈G} is called the orbit of the point X under the action of G. The set of orbits from a set X under the action of a group G is a partition of the set X, and it is known as the quotient set of the action, denoted by X/G.

### 2.3. Four-Klein group

Herein, we develop a novel and logically equivalent approach, where fewer algebraic properties are required, to that followed in our previous works [16,21,24] in which a group structure in the set N = {C, U, G, A} of the four nucleotides was defined. Herein, the ordering of the nucleotides and their arbitrary binary assignments are no longer necessary. A group is naturally constructed with the two types of mutations, transversions and transitions, represented by *a* and *b*, respectively. These two types of transformations are used like generators of the group with the property that the composition (denoted by ○) of a mutation with itself is equal to the identical mutation. The new approach starts with the symmetry group that corresponds to an abstract rectangle, which in group theory is known as the Four-Klein group, here symbolized as $({\text{K}}_{4},\circ ),$ where ${\text{K}}_{4}=\{e,a,b,ab=ba\}$ is the set, and ∘ is the group operation (table 1). The Four-Klein group is identified as an abelian group in the direct product $Z_{2}\times Z_{2},$ where $Z_{2}=\{0,1\}$ represents the cyclic group of two elements. The set $Z_{2}\times Z_{2}$ is regarded as the set of the four duplets of zeros and ones.

**Table 1.** The multiplication table of the Four-Klein group (K_4_,○).

○	*e*	*a*	*b*	*ab*
*e*	*e*	*a*	*b*	*ab*
*a*	*a*	*e*	*ab*	*b*
*b*	*b*	*ab*	*e*	*a*
*ab*	*ab*	*b*	*a*	*e*

### 2.4. Group action in the set of nucleotides

Herein, the set of nucleotides N and its mutations will be considered. The Four-Klein group that will act over the set N, making it mutate, just as a rectangle is transformed in itself through its symmetries. This is represented as the Cayley graph of the group with the nucleotides as vertices. As an example consider the following: a∗(A)=U, a∗(G)=C, b∗(A)=G, b∗(U)=C, while (a∘b)∗(A)=(b∘a)∗(A)=C, and (a∘b)∗(U)=(b∘a)∗(U)=G. For the sake of simplicity, the symbols ∘,∗ and the parentheses will be here and further omitted where no misinterpretation can be made, so that (a∘b)∗(A)=abA.

Now we extend our nucleotide level group action to the set of 64 triplets, N × N × N = N^3^ as follows: $f:{\text{K}}_{4}^{3}\times {\text{N}}^{3}\to {\text{N}}^{3},((a_{1},a_{2},a_{3}),(x_{1}x_{2}x_{3}))\to (a_{1}x_{1},a_{2}x_{2},a_{3}x_{3}),$ where we have used the vector notation and *f* is well defined because the mapping is component-wise.

A common classification of the nucleotides can be done through their chemical properties [24]. Herein, we consider purines and pyrimidines represented as R and Y, respectively, where R = {A, G} and Y = {C, U}. Next, we will deal with codons, which in set notation are the sets: RNY, YNR, YNY and RNR.

### 2.5. Defining a metric or distance in N^3^

For a given choice of generators, one has to define a metric, i.e. the natural distance on the Cayley graph. Here, we have the group K_4_ and its two generators *a* and *b*. The metric is defined in the following manner for *x*_1_, *x*_2_ in N, for single nucleotides:

1. d(*x*_1_, *x*_2_) = 0. If and only if $x_{1}=ex_{2}$.
2. d(*x*_1_, *x*_2_) = 1. If and only if $x_{1}=ax_{2}$ or $x_{1}=bx_{2}$.
3. d(*x*_1_, *x*_2_) = 2. If and only if $x_{1}=abx_{2}=bax_{2}$.

This is a discrete metric that is similar to the one known as Hamming distance, but here the distance is given by the minimum number of generators of the group that are used to take one nucleotide and mutate it into another one. An extension in the definition of distance is natural for triplets so that it will be the sum of the distances of the nucleotides that conform the triplet. Formally, for two triplets *x*_1_*y*_1_*z*_1_ and *x*_2_*y*_2_*z*_2_, the distance is: $\text{d(}x_{1}y_{1}z_{1},x_{2}y_{2}z_{2}\text{)}=\text{d(}x_{1},x_{2}\text{)}+\text{d(}y_{1},y_{2}\text{)}+\text{d(}z_{1},z_{2}\text{)}.$

**Figure 1.**
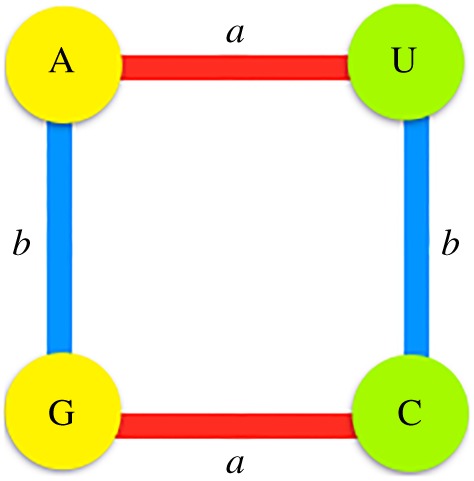
Representation of the action of the generators of the group over the set of nucleotides, where *a* represents transversions and *b* transitions. Purines are coloured in yellow and pyrimidines in green.

The genetic code is then represented as a 6D hypercube. This geometric figure can also be interpreted as a graph *G* = (*V*, *E*) of vertices, representing the codons, and edges, joining the codons at distance one, making it possible to analyse its symmetries through the group of automorphisms of the graph. This group consists of all the bijective functions of the graph G, f: (V,E)→(V,E) that preserve its adjacencies. With the metric defined above, these automorphisms comprise all the isometric transformations of the cube. It is worth mentioning that there are, in essence, only three different Cayley graphs that determine the action of the group over the nucleotides. The pairs of opposite edges of the graph chosen here (figure 1) represent the generators of the group (transversions and transitions), which is in agreement with a common evolutionary interpretation [51]. In our previous approach [16,21,24], the distance of a codon and its anticodon in the 6D hypercube is at the maximum distance of 6. It is worth remarking that, if the Cayley graph associated with our previous works is used, the interchange of the action *a* for *ab*, and *ab* for *a*, applied as described above, will result in the same conclusions. Hence, the two approaches do not contradict each other, neither in biological aspects nor in mathematical ones, owing to the fact that with the present approach the ordering of nucleotides and arbitrary binary assignments are not required. In fact, the four nucleotides A,C,G,U can be situated at the vertices of a given rectangle in 4! = 24 ways. Interestingly, the assumption that *a* and *b* represent transversion and transition, respectively, being *a* the transversion that converts each nucleotide into its complementary, reduces all the possible graphs to only three.

## 3. The Rodin–Rodin model

In the original proposal made by Rodin & Ohno [49], the table of the genetic code is arranged in such a manner, that complementary codons appear vis-à-vis each other. Each of the 20 different aaRSs recognizes the cognate amino acid, and then attaches it to isoacceptor tRNAs with the corresponding anticodons. The operational code provides virtually errorless aminoacylation of tRNAs [6,12,13]. The 20 aaRSs are divided into two 10-member non-overlapping classes, I and II, that have virtually nothing in common with each other as far as the primary sequence, secondary elements and 3D structures are concerned [8].

In their table, amino acids of class aaRS I are coloured in red, while those of class aaRS II are coloured in black (table 2). The amino acids from the first column of the code table tend to belong to class I (Phe being the only exception), whereas the amino acids from the second column all belong to class II.

**Table 2.** Symmetric table of the SGC that is biologically incorrect.

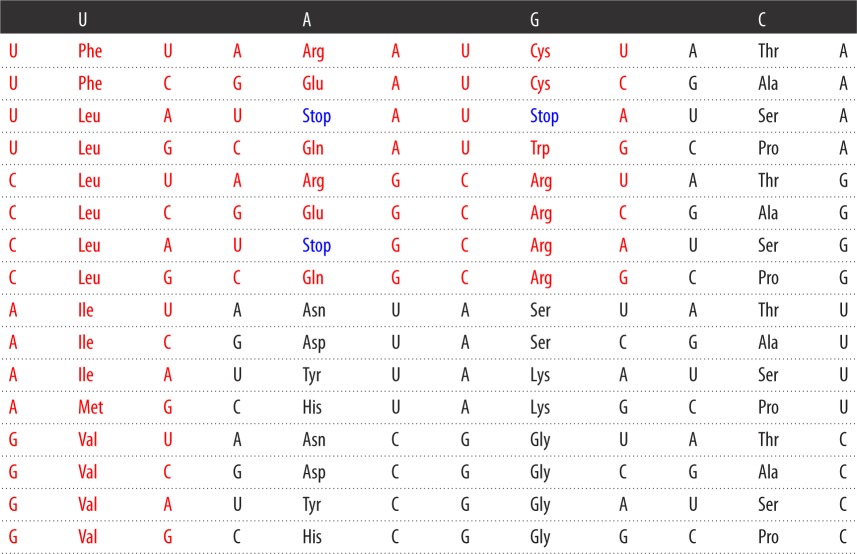

### 3.1. A remarkable observation: a flaw in table 2

In table 2, there is a flaw, which conspires against the symmetries. Lysine and arginine are incorrectly placed. In arginine, two (AGA and AGG) out of its six coding triplets are incorrectly assigned to lysine, whereas the two triplets of lysine, AAA and AAG are assigned to arginine. Rodin & Rodin [50] and Rodin & Ohno [52,53] corrected table 2 [7,49], which is biologically correct but it is not symmetric (see table 3).

**Table 3.** Biologically correct table of the SGC that is not symmetric. Phe and Tyr are ambiguous and they are marked with an asterisk.

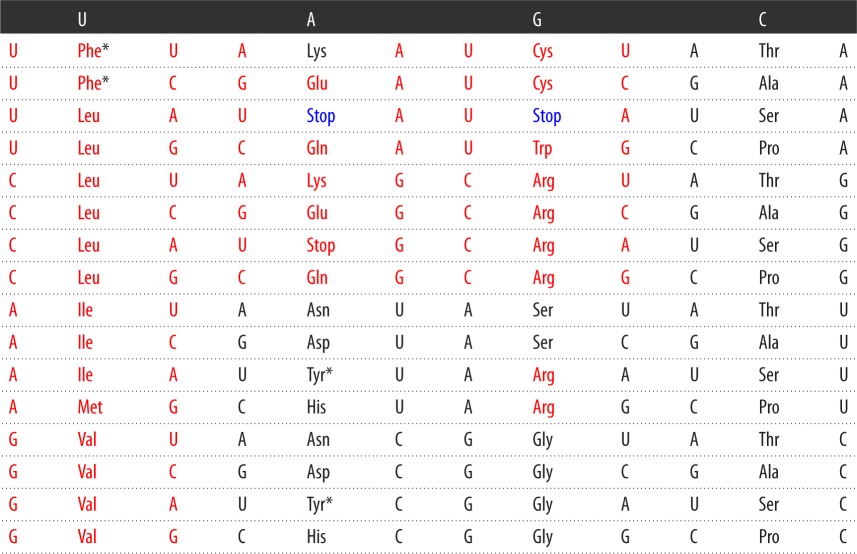

### 3.2. An automorphism that converts the class aaRS I into the class aaRS II and vice versa

**Figure 2.**
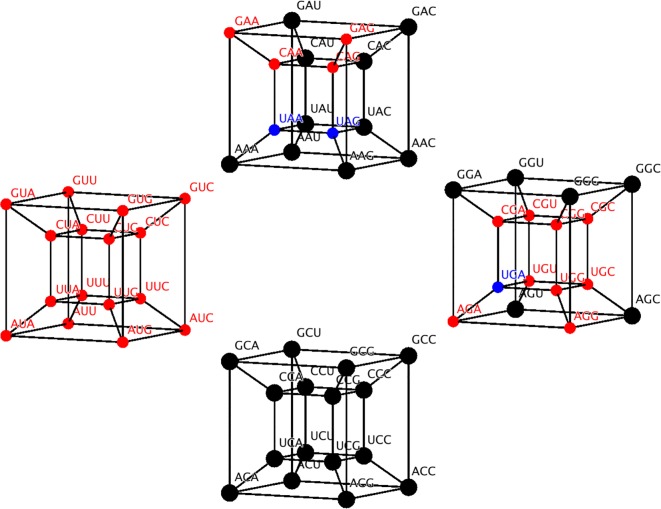
The six-dimensional cube of the genetic code coloured according to the aaRS class, class I is red and class II is black and bold. Stop codons (UUA, UAG and UGA) are in blue although the known cases of their ‘capture’ by amino acids are mostly from class I [52]. The edges joining the four-dimensional cube are not shown for better appreciation.

The RO corrected table of codons associated to each class of aaRS lost symmetry, but in the 6D model this symmetry is recovered. Symmetries are represented with automorphisms of the cube that interchange the codons of class I with class II and vice versa. In fact, there are two such functions, *T*_1_ and *T*_2_ defined piece-wise (table 4). These automorphisms form a subgroup that under composition yields a class invariant transformation $\left(T_{1}\circ T_{2})=T_{3}=(e,e,b\right)$, which is a transition in the wobble position. In figure 2, the codons in the 6D model are coloured according to the aaRS class as in table 2 and table 3 but black is replaced by blue. Each isolated cube is actually a four-dimensional (4D) cube and the union of all of them with their respective extra edges forms the complete 6D cube. The edges joining each 4D cube are omitted for a better appreciation of the complete figure.

**Table 4.** The automorphisms used in each subcode of the SGC to interchange the aaRS classes.

$\_\_\_\_\_\;{\text{T}}_{1}\;\_\_\_\_\_$	$\_\_\_\_\_\;{\text{T}}_{2}\;\_\_\_\_\_$
$\text{RNY}\underset{\left(a,b,a\right)}{↔}\text{YNR}$	$\text{RNY}\underset{\left(a,b,ab\right)}{↔}\text{YNR}$
$\text{RNR}\underset{\left(e,b,e\right)}{↔}\text{RNR}$	$\text{RNR}\underset{\left(e,b,b\right)}{↔}\text{RNR}$
$\text{YNY}\underset{\left(e,b,e\right)}{↔}\text{YNY}$	$\text{YNY}\underset{\left(e,b,b\right)}{↔}\text{YNY}$

### 3.3. From the RO-model to the standard genetic code

According to the RO-model [49], the table of the genetic code can be divided into the sub-codes NAN, NGN, NUN, NCN. There exists an automorphism *F* of the cube defined also piece-wise, which transforms that division into the sub-codes RNR, YNR, RNY, YNY, respectively (table 5), which is precisely our algebraic model [16,21,35]. As an example, consider the codon AGC in the RO-model. AGC is an element of the RGY subcode, so the action required to transform it to our 6D model is (*a*,*ab*,*a*) as described in table 5. From the definition of the group action, this codon will be transformed to the triplet UUG. Note also that, owing to the order of the elements of the group, the same action over UUG on the 6D model will send it back to AGC in the RO-model.

**Table 5.** Automorphisms to convert the Rodin–Ohno model partitions of the genetic code into the RNR, RNY, YNR, YNY partitions.

_____ F _____	_____ F _____
$\text{RAR}\underset{\left(e,e,e\right)}{↔}\text{RAR}$	$\text{RGR}\underset{\left(a,b,e\right)}{↔}\text{YAR}$
$\text{YGR}\underset{\left(e,e,e\right)}{↔}\text{YGR}$	$\text{YAR}\underset{\left(a,b,e\right)}{↔}\text{RGR}$
$\text{RUY}\underset{\left(e,e,e\right)}{↔}\text{RUY}$	$\text{RCY}\underset{\left(a,b,e\right)}{↔}\text{YUY}$
$\text{YCY}\underset{\left(e,e,e\right)}{↔}\text{YCY}$	$\text{YUY}\underset{\left(a,b,e\right)}{↔}\text{RCY}$
$\text{RUR}\underset{\left(e,a,a\right)}{↔}\text{RAY}$	$\text{RCR}\underset{\left(a,ab,a\right)}{↔}\text{YAY}$
$\text{YCR}\underset{\left(e,a,a\right)}{↔}\text{YGY}$	$\text{YUR}\underset{\left(a,ab,a\right)}{↔}\text{RGY}$
$\text{RAY}\underset{\left(e,a,a\right)}{↔}\text{RUR}$	$\text{RGY}\underset{\left(a,ab,a\right)}{↔}\text{YUR}$
$\text{YGY}\underset{\left(e,a,a\right)}{↔}\text{YCR}$	$\text{YAY}\underset{\left(a,ab,a\right)}{↔}\text{RCR}$

## 4. The polar requirement in the six-dimensional SGC

**Figure 3.**
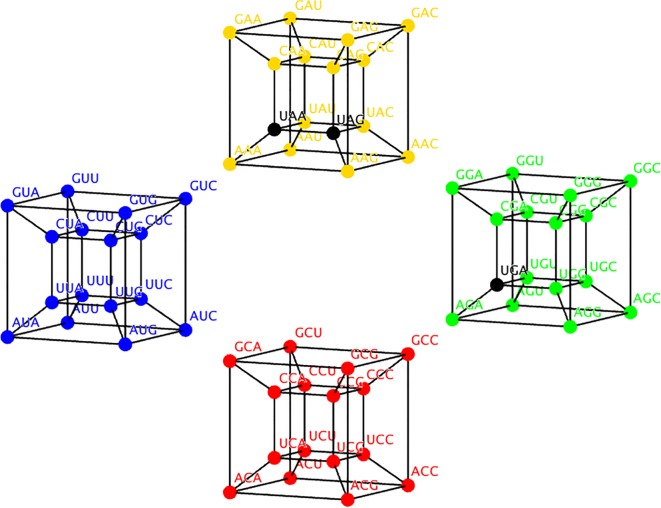
Six-dimensional hypercube of the SGC coloured by amino acid polar requirement values [41]. The four-dimensional hypercubes are yellow (upper); blue (left); red (lower); green (right); Stop codon are in black (UUA, UAG and UGA).

PR was scaled into four categories [41]. We assign a particular colour (red, yellow, blue and green) to each scale. When such categories are set on the 6D genetic code, new symmetries emerge (figure 3). Now the SGC in six dimensions can be symmetrically divided into four colours according to the PR. Each category, or colour, comprises 16 codons that are arranged in 4D hypercubes, whose symmetry is given by the wreath product *S*_2_Wr*S*_4_, where *S_n_* is a permutation group of *n* elements [54]. Such group can be represented by the group of orthogonal matrices of 4 × 4 whose entries are all integers [54]. To interchange whole categories, it is sufficient to use the symmetries of a square $Dih_{4}$ (figure 3). Hence, the 6D representation of the SGC can reflect this property using its automorphisms as a biological classifier.

### 4.1. Delarue's model

Delarue [3] argues that the partition of codons according to the aaRS class distinction facilitated a hierarchical process by which additions to the code reduced codon ambiguity to produce the extant table with just five binary choices. The code started with undifferentiated and nonsense triplets, NNN. Codons were given meaning beginning with the second base and ending with the third. The NYN triplets could interact with a synthetase, whereas the NRN could not and remained stop codons. At each step, the ambiguous codon family differentiated to give descendants with opposite groove recognition, while descent of the stop codon family generated a new ambiguous family and retained a stop codon, which was always present in the code. These asymmetric division rules provide a unique differentiation order, rendering the exhaustive exploration of the initial assignment of codons plausible, and suggesting that the appearance of the code conferred meaning successively from redundancy by a deterministic elimination of the most frequent errors. Notably, tRNAs with complementary anticodons also have statistically significant complementarity in their acceptor-stem operational codes [3].

With the concept of group action in mind, it is possible to analyse the D-model and elaborate an algebraic model. As the order 2 subgroup *T* generated by *b* is the group of transitions of the set N, *T* = {*e, b*} is isomorphic to the cyclic group $Z_{2}.$ The quotient N/*T* represents precisely the partitions {R, Y} of the set of nucleotides. Considering the quotient N/*e*, where *e* is the trivial group, we obtain the nucleotides separated in different sets: N/e={{A},{G},{U},{C}}. Finally, the quotient with the entire group is a trivial operation, with only one class, as N/K_4_ = N. In order to analyse triplets, a component-wise operation naturally arises from these definitions resulting in: NNN/GGG=N/G×N/G×N/G, where *G* is any subgroup of K_4_, i.e. analysing the quotients component by component and then relating them with the cartesian products of sets. Now the Delarue's model given by six binary choices can be algebraically analysed. The quotient $\text{NNN}/{\text{K}}_{4}TK_{4}=\{\text{NRN},\text{NYN}\},$ where *T* = {*e,b*} is the subgroup of transitions, yields the first binary choice and for the next steps doing $\text{NRN}/{\text{K}}_{4}e{\text{K}}_{4}$ and $\text{NYN}/{\text{K}}_{4}e{\text{K}}_{4},$ respectively, and for the rest what is only needed is to use as quotients the products: $Te{\text{K}}_{4},$ $ee{\text{K}}_{4},eeT$ and *eee* in that order. We have just replaced the six binary decisions (including the wobbling assignments) in the D-model by six algebraic well-defined mathematical representations. The value of the latter is that we can follow the groups of symmetries in each step. Furthermore, we can make the model parsimonious and simpler, if we now make quaternary decisions so that the nucleotides in each position of the codon are determined at each step, by the use of only three group products, ${\text{K}}_{4}e{\text{K}}_{4},$ $ee{\text{K}}_{4}$ and *eee*.

## 5. Discussion

In this work, we have been able to formulate algebraic expressions for two well-known models of the origin and evolution of the genetic code, to wit, the RO-model and Delarue's model. Both models are consistent with the RNY code [9], as partitioning of aaRSs in two classes could have been encoded in a strand-symmetric RNA world [7,50]. We have shown that by assuming both a primeval RNY code and that the code can be divided into two classes of the aaRSs, we arrive at a symmetrical representation of the genetic code in a 6D algebraic model. We have also shown that PR displays a symmetrical pattern in this 6D model. PR is an empirical scale unrelated to either of the two transfer equilibria that best represent the partitioning of amino acids between pure phases, rather than between a pure phase and cellulose. PR seems to be also unrelated to other measures such as hydropathy. Further experimental work is needed to clarify these issues.

The aaRSs are a prime example of horizontal gene transfer [55,56]. Evolutionary replacements of aaRSs accompanied the evolution of the genetic code [31]. The assignments seemed to minimize errors in a primitive translation mechanism that was highly inaccurate [57,58]. The evolutionary phylogenies of synthetases do not obey the basic division of all life into the three primary groupings Bacteria, Archaea and Eukaryotes [56]. The two aaRS classes are presumably the oldest protein superfamilies. The RO hypothesis [52] implies that they arose at nearly the same instant in geological time because, at the nucleic acid level, the information necessary for function of each class is indistinguishable from that necessary for function of the other [40]. Complementarity means that one strand implies the existence of the other. Sense/antisense coding thus projects back past the genetic coding nexus to chemistry. The sense/antisense ancestry of the aaRS appears to be solidly established [40,59]. The authors, Rodin & Ohno, observed that their model is *almost perfectly symmetric* [49,52,53]. But in front of this unusual assertion we argue that something that is almost perfectly symmetric is not symmetric at all. Interestingly, the automorphisms *T*_1_ and *T*_2_ show the so-called symmetry that only exists in our 6D model, and the function *F* converts the partitions of the RO-model {NUN, NAN, NGN, NCN} into the partition {RNR, RNY, RNY, YNR}*,* which corresponds to our symmetric model. As the functions presented are isometric, the RO-model may be considered as equivalent to this one and it only takes a different point of view of the same model to reach one's conclusions from the other. The D-model is a phenomenological model of progressive differentiation-like reduction of codon ambiguity [60]. Indeed, it has been suggested that the primitive ribosome worked to synthesize peptides randomly, without the need of a code [61]. This elegant model is also based on the pattern of tRNA aminoacylation by class I and II aaRSs. However, in contrast with our complementarity-based model, Delarue's asymmetric model consists of a binary decision tree, like in a longitudinal differentiation process [3]. The whole SGC is derived from binary decisions but it remains unclear why the minor or major groove side is preferred in each particular step. We propose an algebraic model that accounts for the simultaneous selection of pairs of complementary triplets following the RO-model, and a set of six algebraic well-defined algebraic operations that account for the six binary decisions of the D-model. We have shown that the D-model can be built from simple operations of action groups. The preservation of symmetries is noteworthy. With only two transformations, we can derive, from a single codon, the 32 triplets forming the RNY and YNR subsets, as well as the 32 triplets comprising the sets RNR and YNY. All the transformations required for the construction are subgroups of ${\text{K}}_{4}^{3}$ which is the general group acting on the codon space, therefore making impossible the creation of new codons without a symmetry breaking which is the action of a new subset of operators.

Until now, participation of two aaRS classes in genetic coding has been rationalized as a result of successive binary choices [3] or as a means of avoiding coding ambiguity [60]. It has been shown that this distinction appears to be related to the complementary roles of class I and II amino acids in protein folding. Members of subclass IA (Leu, Ile, Val and Met) have aliphatic side-chains and are found in hydrophobic cores. Members of subclass IIA (Ser, Thr and His) are small amino acids with water-favouring side-chains. Subclasses B (with carboxyl, amide, primary amine side-chains) and C (aromatic) in both classes contain similar amino acids. Class I amino acids tend to be buried; those in class II remain largely on the surface. Class I amino acids allowed formation of non-polar cores and class II amino acids populated the surfaces of globular proteins. The linkage between classes arising from their sense/antisense ancestry [38,62] would be expected to simplify the search for reduced amino acid alphabets that may have been used during early protein evolution, leading to the universal genetic code. The order in which predictors emerge in the stepwise regressions discussed above is similar, but not identical to, the series of decisions by which Delarue suggested that genetic coding actually became fixed [3]. Although tRNA identity elements have probably been confounded by horizontal gene transfer [32], ancestral tRNA sequence reconstruction may clarify further how identity elements and the synthetase class recognition evolved.

With our approach, we have shown that the whole SGC can be derived starting from a pair of reverse complementary codons with just six steps or just three if we follow quaternary decisions. The present algebraic approach is general and abstract enough as it deals with the algebra from outside of the genetic code making it possible to build bridges among different models. This approach permits the direct comparison of different genetic models that otherwise would be difficult to perform. For example, the self-referential (SR) model for the formation of the SGC [14] is appealing because it considers a self-modifying genetic code that alters its own instructions while it is evolving. Consequently, the instruction path length is reduced and improves its performance and maintenance through the mechanism of natural selection. It is called SR because it is centred on the integration of self-feeding ribonucleoprotein structures where the protein and RNA activities are mutually stimulatory, after having been formed on top of the basic tRNA dimers. It assumes that during early stages of the formation of the SGC, protein synthesis was directed by tRNA dimers. The SR-model lacks experimental support but it is compatible with the appearance of the metabolic pathways [63]. The proposed dimer-directed transferase activity should be experimentally tested, either utilizing present-day tRNAs or the various kinds of mini-tRNAs that have been used as acceptors for the aaRS function or for spontaneous aminoacylation. The genetic eukaryotic anticode comprises 46 anticodons as there are not anticodons ending with adenine $\left(3^{\prime }\to 5^{\prime }\right)$ direction. The group actions required to describe the symmetries of this model are given by the direct product $K_{4}\times K_{4}\times Z_{3},$ where the last set is the cyclic group of three elements that corresponds to the rotations of a triangle. The cyclic groups are generated with one element so the biological interpretation of this action is ambiguous, in contrast with the generators of *K*_4_ representing transitions and transversions. Another difference is that this model can only be fully described in five dimensions. These differences in the mathematical properties of the SR-model with our 6D model show that they are non-equivalent and that there is no smooth way to mathematically complete the SGC. Essentially, the problem lies in the fact that the group *K*_4_ cannot be obtained from $Z_{3}.$ The SR-model lacks an explanation of how the dinucleotides formed the codons. Did they appear gradually? Or did codons appear simultaneously from a given set of principal dinucleotides? The chronology of appearance of codons is absent.

The partition of the table of the genetic code into the two classes of aaRSs is entirely consistent with the complementary symmetry of the RNA world in general, and the hypothesis of its initial double-strand coding in particular. It has been shown that the elimination of any amino acid encoded by the primeval RNY code would be strongly selected against and therefore at this stage the RNY code was already frozen [18]. The very existence of the ying-yang (formerly dubbed ‘ying-yang-*like*’ [7]) pattern of aminoacylation that certainly has little if anything at all to do with the present-day protein aaRSs, points to the ‘anticodon first’ scenario of the genetic code origin [64,65]. The anticodon is indeed essential for 17 of the 20 *Escherichia coli* isoaccepting groups [66]. The second operational code does not make sense without the anticodon code. However, the early relevance of the acceptor mini-helix in evolutionary development of the tRNA molecule cannot be understated [13,36,52,59,67,68]. Consistent with the hypothesis that the acceptor double-stranded stem is older than the anticodon loop, the GC-biased codon–anticodon-like triplet pairs located just next to the 73rd base-determinator of the acceptor stem may better reflect the very initial shaping stage of the genetic code than the single-stranded anticodon [50,53].

We have developed mathematical models for the RO-hypothesis and the D-genealogy. We highlight that these mathematical models are different despite the fact that they share the fundamental fact that the SGC can be divided by the two classes of aaRSs. We emphasize that our 6D model is completely equivalent to the mathematical model of the RO-hypothesis. The mathematical model of the SR-hypothesis underscores the differences with the other three models. The 6D symmetrical model has been enriched by the RO-model and the RO-model has acquired a sound mathematical structure. All presented models deal with the same biological aspects of the SGC, but differently. The 6D structure has been exploited not only for comparing different models but more importantly to give a step forward to unify models and reinforce (or weaken) models' hypotheses.

In conclusion, the most adequate model for the SGC can be represented in a 6D hypercube. Each dimension describes a type of mutation, transition and transversion as given by the Cayley graph, acting on each of three bases of any codon. Consequently, we obtain the six dimensions. When considering the hydropathy scale of amino acids [69], there are no symmetries that would interchange the four categories. However, if the codon UGA were associated with an amino acid that falls into the category of ‘moderately hydrophobic’, then the transformation (*e, e, b*) would be invariant to the hydropathy classes. In the same manner, when considering the polarity of amino acids [37–39], it would be needed that UGA were a non-polar amino acid, the transformation (*e, e, b*) would be invariant to polarity. If, in addition, the other stop codons are assigned to polar amino acids, the transformation (*e, e, a*) would be another invariant symmetry, as well as their composition. This means that a biological classification can also be interpreted as symmetries that would maintain the classification.

Undoubtedly, the 6D description of the genetic code as the hypercube ${\left(Z_{2}\right)}^{6},$ becomes essential for a better understanding of the evolution of the code. The SGC, as derived from the primeval genetic code, and the RO-model are one and the same. We have shown that these different models of the genetic code are mathematically equivalent. Hence, the 6D algebraic model presented here unifies different models of the genetic code.

## Data accessibility

The values of polar requirement were taken from [41]; the hydropathy scale of amino acids were taken from [69]. The rest of the work is essentially theoretical.
